# Ridehailing and alcohol-involved traffic fatalities in the United States: The average and heterogeneous association of uber

**DOI:** 10.1371/journal.pone.0238744

**Published:** 2020-09-11

**Authors:** Noli Brazil, David Kirk

**Affiliations:** 1 Department of Human Ecology, University of California, Davis, California, United States of America; 2 Nuffield College and Department of Sociology, University of Oxford, Oxford, United Kingdom; Tongii University, CHINA

## Abstract

Ridehailing services such as Uber have been promoted as viable interventions for curbing alcohol-involved driving fatalities. However, evidence of ridehailing’s impact has been mixed, with some studies finding no association but others finding either an increase or a decrease in fatalities. We contribute to this literature by examining more recent years of data, which capture a period during which Uber ridership has grown substantially and alcohol-involved fatalities have increased. Furthermore, we test whether the relationship between Uber availability and traffic fatalities depends on local characteristics. We employ multivariate regression models to test the association between Uber availability and total, alcohol-involved, and weekend and holiday-specific traffic fatalities in the 100 most populated metropolitan areas in the United States between 2009 and 2017. We find that Uber availability is not associated with changes in total, alcohol-involved, and weekend and holiday-specific traffic fatalities in aggregate, yet it is associated with increased traffic fatalities in urban, densely populated counties.

## Introduction

Alcohol-involved driving is the greatest single cause of motor vehicle deaths in the United States. In 2018, alcohol-involved driving fatalities accounted for 29 percent of traffic deaths, representing a total of 10,511 lives lost [1]. Furthermore, after decades of progress, the number of alcohol-involved driving fatalities stagnated before increasing in recent years, rising 5.7% from 2014 to 2018 [1].

To address this troubling trend, the National Academies of Sciences, Engineering, and Medicine (NAS) recently convened a committee to identify promising strategies to reduce alcohol-involved driving fatalities. Among a suite of recommendations, the committee recommends that municipalities “support policies and programs that increase the availability, convenience, affordability, and safety of transportation alternatives for drinkers who might otherwise drive. This includes permitting transportation network company ridesharing” [2, p.17]. Indeed, the use of smartphone-enabled taxi-type services, commonly referred to as ridehailing or ridesharing, has been widely promoted as an alternative to driving after consuming alcohol because of their added convenience and often lower costs compared to traditional alternative transportation options. Uber, the largest ridehailing company in the United States, partnered with Mothers Against Drunk Driving in 2015 to promote the usage of ridehailing as a way to curb alcohol-involved driving [3]. At the time this partnership formed, the relationship between ridehailing and alcohol-involved fatalities was largely untested, but several studies in the interim have begun to provide initial evidence about this relationship.

For instance, a 2016 study [4] examined the association between Uber availability and traffic fatalities in the 100 largest metropolitan areas in the United States, during the initial five-year period following the advent of ridehailing. In contrast to the common intuition at the time, the authors found that Uber availability was not associated with changes in total, weekend- and holiday-specific, and alcohol-involved traffic fatalities. Since their publication, scholarly interest in understanding the impact of ridehailing has expanded, although the breadth of studies focused on alcohol-involved fatalities is still limited. Across the relatively few studies, evidence of ridehailing’s impact has been mixed, with some studies finding no association but others finding either an increase or a decrease in alcohol-involved and other types of traffic fatalities [5–7].

There are several plausible explanations for an increase in fatalities following the deployment of Uber. For instance, studies have found that ridehailing tends to be a substitute for public transit usage, but it is less clear the extent to which it is a substitute for drunk driving [8, 9]. Because Uber is a substitute for public transit, cities have experienced increasing traffic congestion after Uber deployment, thereby elevating the risk of accidents [9–11]. Moreover, there is even evidence that Uber has increased alcohol consumption and binge drinking [12, 13]. Based on extant evidence, it stands to reason that Uber’s association with alcohol-involved traffic fatalities may depend on ecological characteristics such as population density and public transit access and usage.

There are also reasoned explanations for null findings in prior studies. Some have suggested that ridehailing, during the initial years following inception, may not have penetrated markets strongly enough to produce a significant change in the number of traffic fatalities [4]. In 2014, Uber was available in 88 of the 100 largest metropolitan areas, but only 30 metro areas had Uber services available for more than one year. However, since 2016, Uber has been available in all 100 largest metropolitan areas, and also in hundreds of additional smaller cities and nonurban areas across the United States. Furthermore, whereas it took Uber roughly six years from inception before it reached the 1 billion ride milestone in March 2016, it took just 2.5 more years (to September 2018) to reach 10 billion total rides [14]. Accordingly, the context of ridehailing has changed considerably since the few initial studies of Uber, and so have the trends in alcohol-involved fatalities. Hence, there is a need to revisit and scrutinize the relationship between ridehailing and alcohol-involved fatalities, particularly to confirm whether ridehailing should indeed be a primary focus of public health interventions as the NAS study recommended [2].

In this article, we examine the association between Uber availability and traffic fatalities for the principal counties in the 100 largest metropolitan areas in the United States for the 2009 to 2017 period. Beyond simply answering the question of “whether” Uber is associated with traffic fatalities, we also seek to pinpoint *where* ridehailing might have the most (negative or positive) impact by assessing whether associations depend on local context. For instance, ridehailing may be a solution for drunk driving in dense urban centers, but less effective in suburban environments. We test the moderating association for a battery of variables that measure population size and density, urbanicity, resident demographic and socioeconomic characteristics, vehicle ownership, public transportation access and usage, and alcohol access and consumption.

## Materials and methods

### Data

The sample contains monthly observations between January 2009 and December 2017 for the most populated county in each of the 100 largest metropolitan areas in the United States. To determine the top 100 most populated metropolitan areas in the country, we used the latest delineation of metropolitan areas as defined by the US Census Bureau. We then used 2010 US Census population counts by metropolitan area to rank order these metropolitan areas and constructed our sample based on the top 100 most populated areas [4]. We then selected the largest county in each metropolitan area, with the exception of the New York metropolitan area, which includes the five separate counties that correspond to the five boroughs of New York City (Bronx, Kings, New York, Queens, and Richmond).

The dependent variable is the monthly count of traffic fatalities. We obtained data on total, weekend- and holiday-specific, and alcohol-involved fatalities from the National Highway Traffic Safety Administration (NHTSA) Fatal Accident Reporting System [15]. Alcohol-involved fatalities involved at least one driver whom the police reported alcohol involvement or tested positive for alcohol. Weekend fatalities occurred between 5:00 pm on Friday and 5:00 am on the following Sunday. Holiday-specific fatalities are those that occurred during a major US holiday. We start the observation period in 2009 because of the changes that the NHTSA implemented the year prior in how they recorded alcohol-involved accidents.

**Fig 1:**
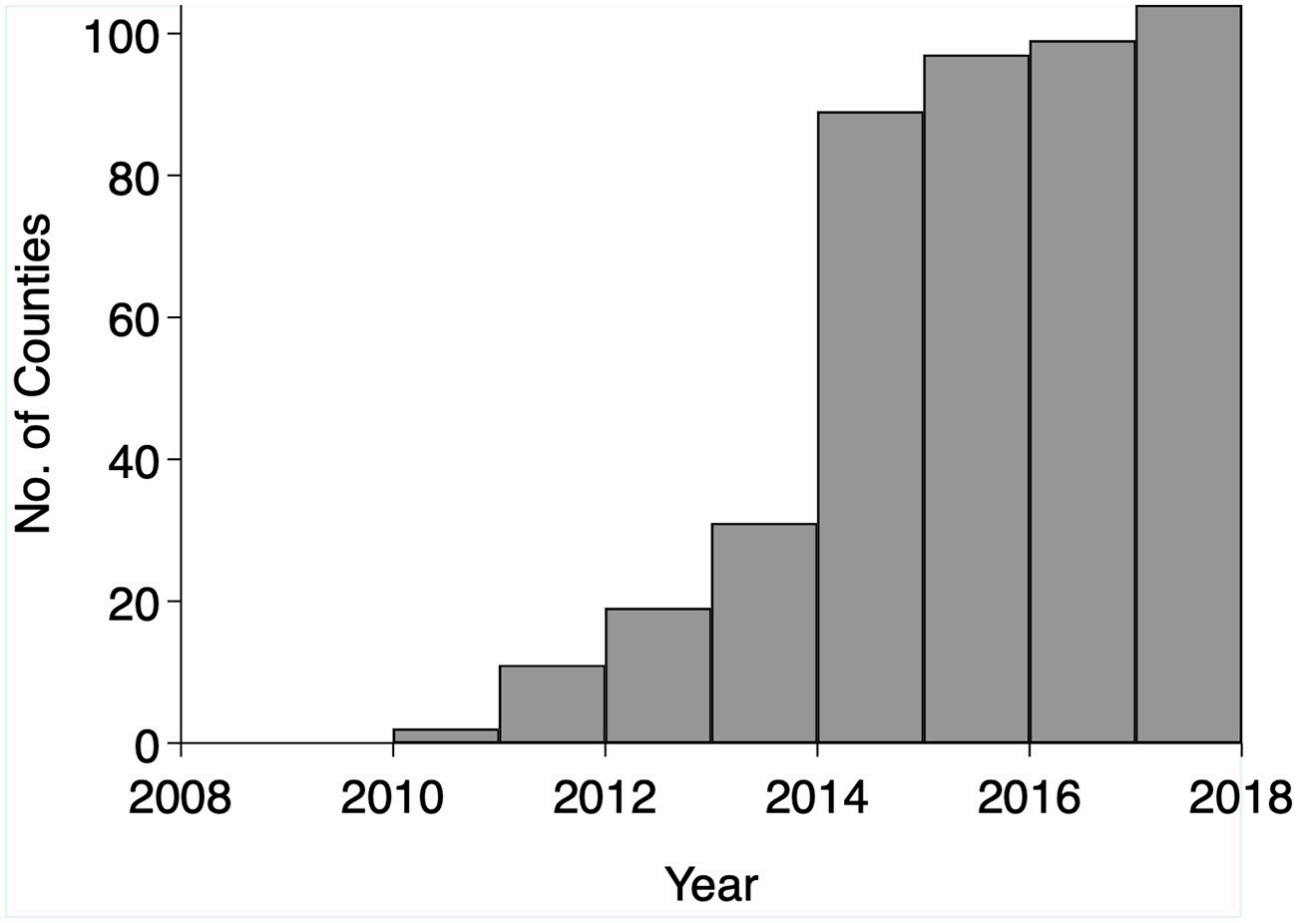
Principal counties in the top 100 largest metropolitan areas in the United States with available Uber services by year, 2009–2017.

Our main independent variable is a binary indicator of whether Uber services were available in a county during a given month and year. We obtained service launch dates directly from Uber. The company defines the start date as the most recent contiguous period where at least 25 active drivers per month are completing trips in a given metropolitan area. Fig 1 shows the number of principal counties in the top 100 metropolitan areas with available Uber services by year between 2009 and 2017. The figure shows that Uber became available for a large proportion of counties in the sample after 2013 (70%).

We gathered data on a variety of county-level characteristics to test the moderating or heterogeneous association of Uber and traffic fatalities. The first set of characteristics captures measures of urbanicity and population mass and density. We included total population, population density, and urban centrality, the last of which is measured as the percent of the county population that lives inside a census designated urban area. Data on these characteristics were obtained from the 5-year American Community Surveys (ACS).

The second set of characteristics measures private vehicle ownership and public transit access and usage. We included the number of available vehicles per housing unit, the percentage of total vehicle miles traveled (VMT) that are attributed to public transit vehicles, the percentage of total VMT that are attributed to rail specific public transit, and measures of public transit coverage and service frequency. Public transit coverage is measured as the share of working-age residents living in block groups with access to at least one transit stop within ¾ mile of their population weighted centroid. Service frequency is measured as the average number of minutes that commuters must wait between bus or train stops. Vehicle ownership data were obtained from the ACS, and rail, total public transit, and total VMT data were obtained from the Federal Transit Administration’s National Transit Database [16]. Transit coverage and service frequency data were obtained from prior work [17].

The next set of variables captures county demographic and socioeconomic characteristics that potentially alters the strength of the relationship between Uber availability and traffic fatalities. We included log median household income, the percent of residents 25 years and over with a college degree, and the percent of residents that are between 20 to 39 years old. Data on these characteristics were obtained from the ACS. The final set of variables captures alcohol access and consumption. We included the number of drinking establishments per county area, where drinking establishments are defined as those with licenses to sell and consume alcohol on the business premises. We also included measures of the percent of adults reporting drinking any alcohol in the past 30 days before being surveyed and the percent of adults reporting binge or heavy drinking in the past 30 days before being surveyed, where binge drinking is defined as drinking 5 or more drinks on an occasion for men or 4 or more drinks on an occasion for women, and heavy drinking is defined as drinking 15 or more drinks per week for men or 8 or more drinks per week for women. Drinking establishment data were obtained from the Census County Business Patterns (CBP) and resident alcohol consumption data were obtained from county-aggregated data from the Behavioral Risk Factor Surveillance System [18, 19].

All moderating characteristics were measured for the year or period prior to Uber entering the county except for public transit service frequency and access, which were measured in 2011 for all counties. For instance, with the exception of the public transit indicators, we used the 2009–2013 ACS to measure moderators for counties deploying Uber for the first time in 2013. For each variable, we split the sample into quartiles based on the sample population, and then categorized counties in the top quartile as high, the bottom quartile as low, and the middle two quartiles as mid.

### Statistical analyses

Because the dependent variable is the number of fatalities, we use a Poisson regression model to estimate the relationship between Uber availability and traffic fatalities. Our measure of exposure is monthly VMT, which we estimated by multiplying the state’s monthly VMT by the county’s proportion of its state’s total roadway mileage. We obtained VMT and roadway length data from the Federal Highway Administration [20]. VMT is a common measure of crash fatality risk because it captures the number of persons exposed to driving over a given time period.

We use an observational panel study design to examine within-county changes in monthly motor vehicle fatalities after the implementation of Uber services. We estimate a regression model of the following form:

$$Y_{ct}=\alpha +\beta {Uber}_{ct}+\theta_{c}+\gamma_{t}+\delta_{c}t+\sigma X_{ct}+\varepsilon_{ct}$$ (1)

where *Y*_*ct*_ is the number of traffic fatalities in county *c* in a given month *t*, *Uber*_*ct*_ is a dummy variable indicating whether Uber was active or not in a county during a given month, *θ*_*c*_ is a county fixed effect, *γ*_*t*_ is a month-by-year fixed effect, *δ*_*c*_*t* is a county-specific linear time trend, and *X*_*ct*_ are controls for monthly unemployment rates and yearly total population, which prior work has shown to predict ridehailing market entrance [7]. We adjust standard errors for clustering at the county level.

To test for heterogeneity, we include interactions between the Uber active indicator variable and the high/mid/low categorization of each moderating variable. Rather than report model coefficients, we estimate the marginal associations of Uber, which provides the association between Uber and fatalities in each high/mid/low category. We conducted all analyses in Stata, version 15 (StataCorp LP, College Station, Texas). All data and Stata code are available on Github.

## Results

### Main analyses

Our sample contains 11,232 county-monthly observations, 4,767 and 6,465 with and without Uber services available, respectively. Table 1 presents the descriptive statistics of our sample, separately for pre and post Uber deployment. Counties experienced more total (8.22 vs. 6.06), alcohol-involved (2.34 vs 1.97) and weekend- and holiday-specific (2.84 vs. 2.11) traffic fatalities during months when Uber was available. Counties with Uber also have greater population sizes, lower unemployment rates, and higher total vehicle miles travelled. Summary statistics of all moderating variables are provided in the S1 Table (S1 Appendix).

**Table 1:** Descriptive statistics for county-month sample, principal counties of the largest 100 US metropolitan areas, 2009–2017.

Variable	County-months with Uber (N = 4,767), Mean (SD)		County-months without Uber (N = 6,465), Mean (SD)	
Traffic fatalities				
Total	8.22	(9.55)	6.06	(6.36)
Alcohol-involved	2.34	(3.14)	1.97	(2.46)
On weekends and holidays	2.84	(3.73)	2.11	(2.67)
Total population	1,271,602	(1,454,550)	917,087	(998,428)
Unemployment rate	5.44	(1.85)	8.46	(2.38)
Vehicle miles travelled	416.79	(510.49)	346.73	(360.41)

Abbreviation: SD, standard deviation.

Table 2 presents results from Poisson models of Uber’s association with traffic fatalities. We present the coefficients as incidence rate ratios. An incidence rate ratio less than 1 indicates a reduction in traffic fatalities after Uber entry, whereas a ratio greater than 1 suggests an increase. Panels are separated by type of traffic fatality, with model 1 representing a null model and model 2 including all controls and fixed effects. Results from model 1 indicate positive statistically significant associations between Uber availability and total and weekend- and holiday-specific fatalities, but no significant association with alcohol-involved fatalities. After including the full set of controls, however, we no longer observe any statistically significant association between the availability of Uber and any category of traffic fatality.

**Table 2:** Incidence rate ratios for the association of uber availability and total, weekend- and holiday-specific, and alcohol–involved related traffic fatalities, principal counties of the largest 100 US metropolitan areas, 2009–2017^a^.

Traffic fatality	Model 1		p	Model 2^b^		p
	IRR	95% CI		IRR	95% CI	
Total	1.13	1.06, 1.20	< 0.001	1.01	0.97, 1.06	0.471
Weekend and Holiday	1.12	1.04, 1.20	0.002	1.03	0.96, 1.11	0.456
Alcohol-involved	0.99	0.92, 1.06	0.721	1.00	0.95, 1.06	0.902

Abbreviations: CI, confidence interval; IRR, incidence rate ratio.

^a^Each model accounts for county monthly vehicle miles travelled

^b^Includes unemployment rate, total population, county and month by year fixed effects, and a county linear time trend

### Sensitivity analyses

We ran a set of additional models to test the robustness of our main results (S1 Appendix). First, we estimated models excluding New York, San Francisco, and Los Angeles, which represent the cities with the largest Uber presence in the country based on ridership and revenue (S1 Table in S1 Appendix). Second, we accounted for the presence of Lyft, Uber’s largest competitor, by testing a variable that indicates whether either company was present in a county (S1 Table in S1 Appendix). Third, we tested for the association of UberX rather than any Uber service (S1 Table in S1 Appendix). UberX is the company’s lowest cost ridehailing option and has been used in lieu of general Uber availability in prior research on traffic fatalities [6, 7, 20]. Uber and UberX start dates generally are the same, but are more likely to differ for counties earlier in the Uber adoption timeline. Fourth, rather than fatalities, we used the number of accidents involving any, weekend- or holiday-specific, and alcohol-involved fatality as the outcome (S1 Table in S1 Appendix). Fifth, we tested a county-specific quadratic time trend to account for nonlinear secular trends influencing the relationship between Uber availability and fatalities (S1 Table in S1 Appendix). Sixth, we included other control variables in the model, specifically state law indicators associated with alcohol-involved driving, metropolitan area taxi drivers per 100,000 persons, and state beer tax rates (S1 Table in S1 Appendix). Seventh, we ran first- (one month lag of the dependent variable) and second-order (two month lag) autoregressive models to account for serial correlation (S1 Table in S1 Appendix). Eighth, to test whether findings were sensitive to distributional assumptions, we fitted negative binomial regression models, for the main effect models (S1 Table in S1 Appendix). Also, rather than a count regression model, we estimated ordinary least squares regression (OLS) models using two different continuous outcome variables: the logarithm of fatality counts, adding one in months where there were zero fatalities, and the number of fatalities per population (S1 Table in S1 Appendix). Because the OLS outcomes are right censored because they cannot go below 0, we also estimated Tobit regression models [21] (S1 Table in S1 Appendix). Results for all of these robustness tests were highly consistent with the main results.

### Heterogeneity analyses

Table 3 presents results of models testing heterogeneous associations by total population, population density, and urban centrality. We find that total population has no moderating influence on any fatality types. However, Uber is generally associated with increased total, weekend- and holiday-specific, and alcohol-involved fatalities for counties with high population density and urban centrality. Focusing on the alcohol-involved fatalities, for counties in the upper quartile in population density, fatalities are 1.09 higher when Uber is available (95% confidence interval: 1.01, 1.18). For counties in the upper quartile on urban centrality, alcohol-involved traffic fatality rates are 1.10 higher when Uber is available. Conversely, for counties in the lowest quartile, alcohol-involved traffic fatalities are 0.89 lower when Uber is available, although in both cases the association is outside the conventional 0.05 threshold of statistical significance.

**Table 3:** Incidence rate ratios for the association of uber availability and total, weekend- and holiday-specific, and alcohol–involved related traffic fatalities by total population, population density, and urban centrality, principal counties of the largest 100 US metropolitan areas, 2009–2017^a^.

Variable	Total		p	Weekend and Holidays		p	Alcohol-involved		p
	IRR	95% CI		IRR	95% CI		IRR	95% CI	
*Total population*									
Low	1.01	0.89, 1.12	0.903	1.01	0.84, 1.18	0.902	1.03	0.83, 1.23	0.774
Mid	0.98	0.94, 1.03	0.507	1.02	0.93, 1.11	0.654	0.96	0.87, 1.05	0.374
High	1.04	0.97, 1.10	0.263	1.04	0.93, 1.14	0.509	1.03	0.95, 1.10	0.458
*Population density*									
Low	1.01	0.95, 1.07	0.786	1.02	0.93, 1.11	0.728	0.93	0.85, 1.01	0.088
Mid	0.99	0.92, 1.05	0.647	0.95	0.86, 1.04	0.269	0.99	0.90, 1.07	0.761
High	1.06	1.01, 1.12	0.027	1.16	1.01, 1.30	0.020	1.09	1.01, 1.18	0.027
*Urban centrality*									
Low	0.98	0.90, 1.06	0.612	1.02	0.89, 1.14	0.803	0.89	0.78, 1.00	0.066
Mid	0.98	0.93, 1.03	0.437	0.94	0.87, 1.01	0.112	0.99	0.92, 1.05	0.682
High	1.10	1.03, 1.16	0.005	1.19^b^	1.05, 1.33	0.004	1.10	0.99, 1.22	0.071

Abbreviations: CI, confidence interval; IRR, incidence rate ratio.

^a^All models account for county monthly vehicle miles travelled and includes unemployment rate, total population, county and month by year fixed effects, and a county linear time trend

**Fig 2:**
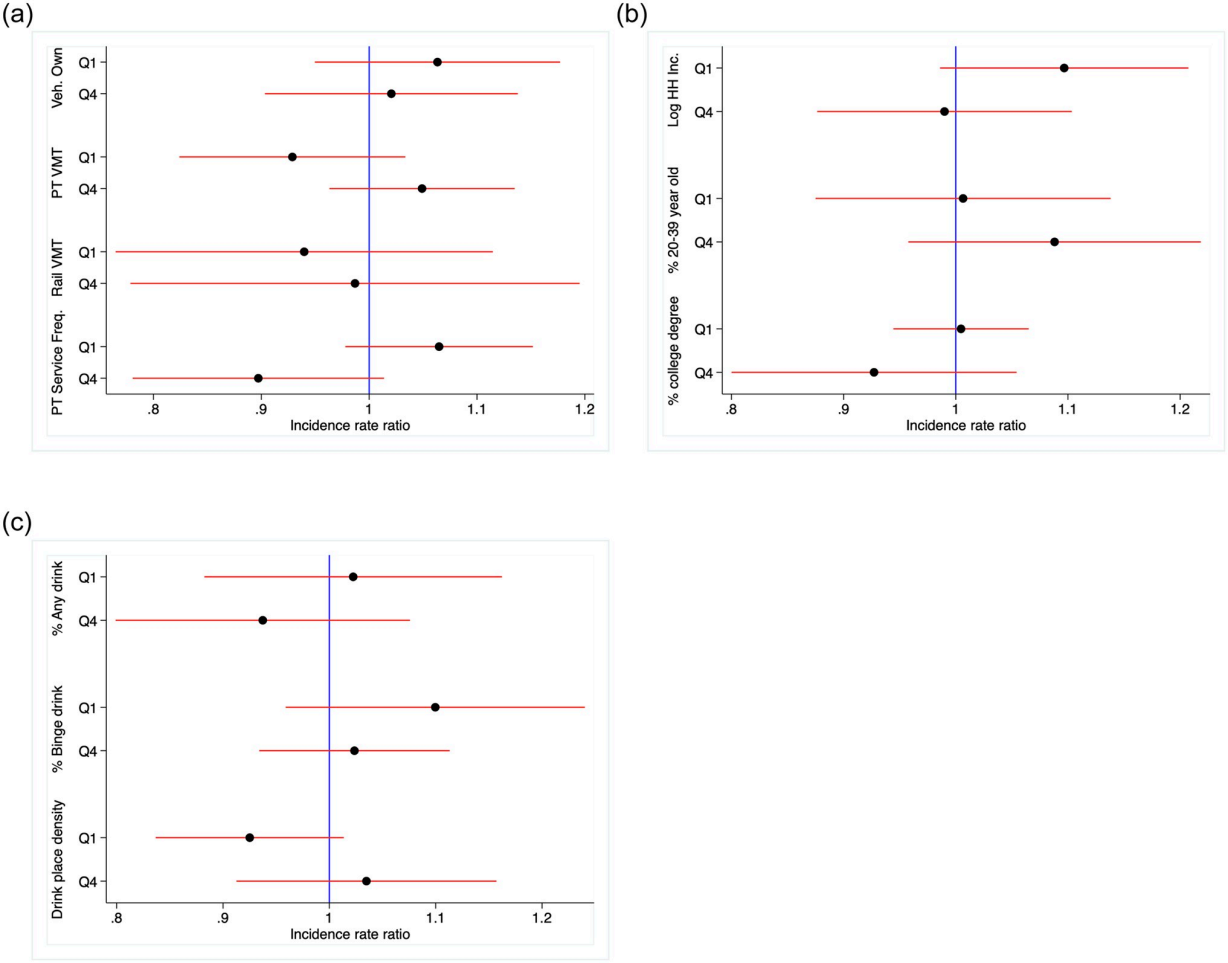
Incidence rate ratios for the association of uber availability and alcohol–involved traffic fatalities by top and bottom quartile on county characteristics, principal counties of the largest 100 US metropolitan areas, 2009–2017. (a) Public transit access and vehicle ownership, (b) Demographic and socioeconomic characteristics, and (c) Alcohol access and usage.

Fig 2 present plots showing the association of Uber separated by our other three sets of moderating variables. Each figure shows incidence rate ratio estimates and 95% confidence intervals for counties in the high (fourth quartile) and low (first quartile) categories of each variable. We focus on alcohol-involved fatalities as they are the main emphasis in the study. Fig 2a shows results for variables related to private vehicle ownership and public transit access and usage. Fig 2b presents estimates for demographic and socioeconomic moderators. Fig 2c presents estimates for alcohol access and consumption. The incidence rate ratios across all models are not statistically significant at the 95% level.

## Discussion

In this study, we extend the relatively limited scholarly focus on the consequences of ridehailing for traffic fatalities by examining more recent years of data that coincide with the recent uptick in national traffic fatality statistics, and by conducting a series of analyses designed to tease apart the question of *where* in particular the availability of Uber might be associated with an increase or decrease in traffic fatalities. We found that, in aggregate, Uber availability exhibits no association with total, weekend- and holiday-specific, and alcohol-involved fatalities. We undertook a variety of sensitivity analyses and found similar results.

We also tested for heterogeneous associations across a set of county-level characteristics measured just prior to Uber’s deployment. We found that while total population size, measures of public transit access, private vehicle ownership, and demographic and socioeconomic characteristics were not moderating factors, Uber availability in counties exhibiting high population density and urbanicity is associated with increases in traffic fatalities, including weekend/holiday fatalities as well as alcohol-involved fatalities.

These results are surprising in that they counter results from previous studies which have largely found either no association or negative associations between ridehailing and traffic fatalities [4–6, 22]. The exception from prior research is Barrios et al.’s recent study of UberX, which found an increase in total traffic fatalities after UberX deployment in U.S. cities [7]. They attributed the increase in traffic fatalities to the increased congestion and road usage caused by Uber availability, an association consistent with other studies of traffic congestion [10, 11]. Another study found an association between ridehailing and increased motorist crash incidences in New York City [23]. The authors also cited increased vehicular congestion as a likely explanatory factor for the increase. Whereas our results diverge from the Barrios’ et al study to some extent, our tests for heterogeneous associations presented in Table 3 corroborate their results by showing that the increased association with fatalities is concentrated in population dense and highly urbanized areas. We go further by examining alcohol-involved fatalities in particular, and observed a worrisome pattern of increases in alcohol-involved fatalities in dense urban centers following the deployment of Uber.

To explain the lack of a main effect of Uber’s availability yet significant interaction associations with population density and urbanicity, we first note that Uber recently released its first ever *Safety Report* [24]. Whereas much of the focus of the report is on documenting the extent of sexual assault occurring in Uber rides and by Uber drivers, Uber went further in the report to investigate the extent of motor vehicle fatalities involving Uber. They find that the rate of traffic fatalities involving an Uber driver, per vehicle mile travelled, is actually half of the rate in the total population [24]. Hence, whereas we find that traffic fatalities, including weekend/holiday as well as alcohol-related fatalities, increased in population dense and urban centered areas, the increases do not necessarily involve Uber drivers. Rather, as Barrios and colleagues suggested, the association could be indirect, such as through the impact of Uber’s service on increases in traffic congestion [7].

Limitations of our research design are relevant to consider. In particular, a limitation common to the entire body of scholarly work on the association of Uber’s service and traffic fatalities is that we lack individual-level information about Uber rides, drivers, and users. Therefore, it is not possible to examine, at the individual-level, to what extent drunk drivers and other high-risk individuals make use of Uber’s services. Similarly, we do not have information on the specific risk profile of Uber drivers (e.g., the extent of sleep deprivation and level of familiarity of the locations where they are driving). That said, the aforementioned Uber *Safety Report* provides some insights about the riskiness of Uber drivers.

A second limitation is related to the U.S.-centric focus of our study. To date, there has been very little research on the association between ridehailing and traffic fatalities focused on countries besides the United States (the few exceptions include recent studies of Chile, Great Britain, and South Africa), yet two of Uber’s top five markets (London and Sao Paulo) are not in the United States and the majority of ridehailing trips worldwide occur outside of the United States, with an estimated 70 percent in Asia alone [25–28]. Hence, future research will need to examine the association between ridehailing and traffic fatalities beyond the United States before a full accounting of the impacts of ridehailing can be determined.

The implications of ridehailing for public safety is a very timely and pertinent topic. If, counter to our findings, further investigation reveals that ridehailing yields declines in alcohol-involved traffic fatalities, then cities might consider how to foster greater use of ridehailing. Conversely, findings presented in our study as well as some other studies suggest that there are potentially serious tradeoffs between the convenience and low cost of ridehailing versus safety [7]. Hence, it would be advisable for policymakers and practitioners to continue to develop a broad base of interventions for curtailing traffic fatalities, and seek to minimize any adverse consequences associated with ridehailing.

## Supporting information

S1 Appendix (DOCX)
