# Supplementary material for: Ridehailing and alcohol-involved traffic fatalities in the United States: The average and heterogeneous association of uber
Source: PLoS One. 2020 Sep 11;15(9):e0238744. doi: 10.1371/journal.pone.0238744 (PMC7485824; doi:10.1371/journal.pone.0238744)
Supplement: S1 Appendix — (DOCX) [file pone.0238744.s001.docx]

**S1 Appendix**

S1 Table 1: Descriptive Statistics of Moderating Variables for County-Month Sample, Principal Counties of the Largest 100 US Metropolitan Areas, 2009–2017

| **Variable** | **Mean** | **SD** |
| --- | --- | --- |
| Total population | 1,038,299 | 1,196,275 |
| Population density | 3,267.61 | 8,536.41 |
| Urban centrality | 94.27 | 4.93 |
| Owned vehicles per housing unit | 1.61 | 0.30 |
| % Total Public Transit VMT | 8.83 | 21.25 |
| % Rail VMT | 6.86 | 18.01 |
| Public transit service frequency | 8.53 | 4.34 |
| Public transit coverage | 63.17 | 18.68 |
| Log median household income | 10.84 | 0.18 |
| % 20-39 year olds | 28.92 | 3.73 |
| % with college degree | 31.58 | 8.00 |
| Drinking places density | 0.75 | 2.88 |
| % of adults reporting any drinking. | 56.74 | 7.37 |
| % of adults reporting binge or heavy drinking | 16.93 | 3.01 |
| Abbreviation: SD, standard deviation; VMT, Vehicles Miles Travelled | | |

S1 Table 2: Incidence Rate Ratios for the Association of Uber Availability and Total, Weekend- and Holiday-Specific, and Alcohol–Involved Related Traffic Fatalities by Principal Counties of the Largest 100 US Metropolitan Areas, 2009-2017, Alternative Modelling Specifications^a^

|  | **Exclude LA, SF  and NY** | | | **Uber or Lyft** | | | **UberX** | | | **Accidents** | | | **Quadratic time  trend** | | | |  |
| --- | --- | --- | --- | --- | --- | --- | --- | --- | --- | --- | --- | --- | --- | --- | --- | --- | --- |
| **Traffic fatality** | **IRR** | **95% CI** | **p** | **IRR** | **95% CI** | **p** | **IRR** | **95% CI** | **p** | **IRR** | **95% CI** | **p** | | **IRR** | **95% CI** | **p** | |
| Total | 1.00 | 0.96, 1.04 | 0.98 | 1.02 | 0.98, 1.06 | 0.36 | 1.01 | 0.97, 1.06 | 0.60 | 1.01 | 0.98, 1.05 | 0.48 | | 1.01 | 0.97, 1.06 | 0.49 | |
| Weekend and Holiday | 0.99 | 0.92, 1.07 | 0.88 | 1.03 | 0.96, 1.11 | 0.39 | 1.01 | 0.93, 1.09 | 0.82 | 1.03 | 0.96, 1.10 | 0.38 | | 1.03 | 0.96, 1.11 | 0.47 | |
| Alcohol-involved | 0.98 | 0.93, 1.05 | 0.61 | 1.00 | 0.95, 1.06 | 0.89 | 0.99 | 0.93, 1.05 | 0.72 | 1.00 | 0.95, 1.06 | 0.94 | | 1.00 | 0.95, 1.06 | 0.92 | |
| Abbreviations: CI, confidence interval; IRR, incidence rate ratio. LA: Los Angeles; SF: San Francisco; NY: New York  ^a^All models account for county monthly vehicle miles travelled and includes unemployment rate, total population, county and month by year fixed effects, and a county time trend | | | | | | | | | | | | | | | | |  |

S1 Table 3: Incidence Rate Ratios for the Association of Uber Availability and Total, Weekend- and Holiday-Specific, and Alcohol–Involved Related Traffic Fatalities, Principal Counties of the Largest 100 US Metropolitan Areas, 2009-2017, Additional Control Variables^a^

| **Variable** | **Total** | | |  | **Weekend and Holiday** | | |  | **Alcohol-Involved** | | |
| --- | --- | --- | --- | --- | --- | --- | --- | --- | --- | --- | --- |
|  | **IRR** | **95% CI** | **p** |  | **IRR** | **95% CI** | **p** |  | **IRR** | **95% CI** | **p** |
| Uber service available | 1.03 | 0.99, 1.07 | 0.22 |  | 1.05 | 0.97, 1.12 | 0.22 |  | 1.01 | 0.96, 1.07 | 0.62 |
| Unemployment rate | 0.98 | 0.96, 1.00 | 0.048 |  | 0.99 | 0.96, 1.01 | 0.24 |  | 0.97 | 0.94, 0.99 | 0.01 |
| State beer tax, 2017 dollars per gallon | 0.99 | 0.88, 1.12 | 0.93 |  | 1.03 | 0.91, 1.16 | 0.67 |  | 1.19 | 0.88, 1.63 | 0.26 |
| Taxi drivers per 100,000 persons | 1.00 | 1.00, 1.00 | 0.25 |  | 1.00 | 1.00, 1.00 | 0.11 |  | 1.00 | 1.00, 1.00 | 0.22 |
| Marijuana decriminalization | 1.16 | 1.06, 1.28 | 0.002 |  | 1.21 | 1.01, 1.45 | 0.04 |  | 1.23 | 1.03, 1.46 | 0.02 |
| Graduated driver licensing law | 1.09 | 1.04, 1.14 | 0.001 |  | 1.14 | 1.05, 1.24 | 0.003 |  | 1.82 | 1.67, 1.98 | 0.00 |
| Hands-free driving law | 1.02 | 0.91, 1.14 | 0.77 |  | 1.08 | 0.83, 1.39 | 0.57 |  | 0.97 | 0.81, 1.17 | 0.77 |
| Medical marijuana legalization | 0.99 | 0.93, 1.05 | 0.73 |  | 0.94 | 0.84, 1.06 | 0.29 |  | 0.93 | 0.84, 1.04 | 0.20 |
| Drug per se law | 0.99 | 0.89, 1.11 | 0.86 |  | 0.88 | 0.72, 1.09 | 0.24 |  | 1.04 | 0.78, 1.39 | 0.79 |
| Seatbelt law, primary enforcement | 1.00 | 0.95, 1.06 | 0.61 |  | 0.98 | 0.90, 1.07 | 0.94 |  | 1.01 | 0.90, 1.13 | 0.95 |
| Ban on texting | 1.00 | 0.95, 1.05 | 0.94 |  | 0.95 | 0.87, 1.03 | 0.20 |  | 0.98 | 0.91, 1.06 | 0.62 |
| Abbreviations: CI, confidence interval; IRR, incidence rate ratio. ^a^All models account for county monthly vehicle miles travelled and includes unemployment rate, total population, county and month by year fixed effects, and a county linear time trend | | | | | | | | | | | |

S1 Table 4: Incidence Rate Ratios for the Association of Uber Availability and Total, Weekend- and Holiday-Specific, and Alcohol–Involved Related Traffic Fatalities, Principal Counties of the Largest 100 US Metropolitan Areas, 2009-2017, Autoregressive First- and Second-Order Lags^a^

|  | **AR(1)** | | |  | **AR(2)** | | |
| --- | --- | --- | --- | --- | --- | --- | --- |
| **Traffic fatality** | **IRR** | **95% CI** | **p** |  | **IRR** | **95% CI** | **p** |
| Total | 1.02 | 0.98, 1.06 | 0.45 |  | 1.02 | 0.98, 1.06 | 0.42 |
| Weekend and Holiday | 1.02 | 0.95, 1.10 | 0.51 |  | 1.03 | 0.96, 1.11 | 0.45 |
| Alcohol-involved | 1.00 | 0.95, 1.06 | 0.98 |  | 1.01 | 0.95, 1.06 | 0.84 |
| Abbreviations: AR, autoregressive. CI, confidence interval; IRR, incidence rate ratio ^a^All models account for county monthly vehicle miles travelled and includes unemployment rate, total population, county and month by year fixed effects, and a county linear time trend | | | | | | | |

S1 Table 5: Incidence Rate Ratios for the Association of Uber Availability and Total, Weekend- and Holiday-Specific, and Alcohol–Involved Related Traffic Fatalities, Principal Counties of the Largest 100 US Metropolitan Areas, 2009-2017, Negative Binomial Regression^a^

|  | **Model 1** | | |  | **Model 2^b^** | | |
| --- | --- | --- | --- | --- | --- | --- | --- |
| **Traffic fatality** | **IRR** | **95% CI** | **p** |  | **IRR** | **95% CI** | **p** |
| Total | 1.16 | 1.08, 1.24 | 0.00 |  | 1.01 | 0.97, 1.05 | 0.58 |
| Weekend and Holiday | 1.15 | 1.07, 1.23 | 0.00 |  | 1.02 | 0.95, 1.10 | 0.57 |
| Alcohol-involved | 0.99 | 0.91, 1.06 | 0.70 |  | 1.00 | 0.95, 1.06 | 0.95 |
| Abbreviations: CI, confidence interval; IRR, incidence rate ratio. ^a^Each model accounts for county monthly vehicle miles travelled ^b^Includes unemployment rate, total population, county and month by year fixed effects, and a county linear time trend | | | | | | | |

S1 Table 6: Regression Coefficients of the Association of Uber Availability and Total, Weekend- and Holiday-Specific, and Alcohol–Involved Related Traffic Fatalities, Principal Counties of the Largest 100 US Metropolitan Areas, 2009-2017, Ordinary Least Squares Regression^a^

|  | **Log (fatal + 1)** | | |  | **Fatalities per population** | | |
| --- | --- | --- | --- | --- | --- | --- | --- |
| **Traffic fatality** | **Coefficient** | **95% CI** | **p** |  | **Coefficient** | **95% CI** | **p** |
| Total | -0.005 | -0.045, 0.034 | 0.79 |  | 0.014 | -0.020, 0.048 | 0.41 |
| Weekend and Holiday | 0.000 | -0.051, 0.051 | 0.99 |  | 0.003 | -0.017, 0.022 | 0.79 |
| Alcohol-involved | -0.002 | -0.046, 0.042 | 0.93 |  | 0.004 | -0.012, 0.019 | 0.62 |
| Abbreviations: CI, confidence interval. ^a^All models account for county monthly vehicle miles travelled and includes unemployment rate, total population, county and month by year fixed effects, and a county linear time trend | | | | | | | |

S1 Table 7: Regression Coefficients of the Association of Uber Availability and Total, Weekend- and Holiday-Specific, and Alcohol–Involved Related Traffic Fatalities, Principal Counties of the Largest 100 US Metropolitan Areas, 2009-2017, Tobit Regression^a^

|  | **Log (fatal + 1)** | | |  | **Fatalities per population** | | |
| --- | --- | --- | --- | --- | --- | --- | --- |
| **Traffic fatality** | **Coefficient** | **95% CI** | **p** |  | **Coefficient** | **95% CI** | **p** |
| Total | -0.009 | -0.049, 0.031 | 0.660 |  | 0.010 | -0.025, 0.044 | 0.580 |
| Weekend and Holiday | -0.004 | -0.069, 0.060 | 0.890 |  | 0.001 | -0.025, 0.027 | 0.950 |
| Alcohol-involved | -0.003 | -0.067, 0.062 | 0.930 |  | 0.003 | -0.021, 0.027 | 0.820 |
| Abbreviations: CI, confidence interval. ^a^All models account for county monthly vehicle miles travelled and includes unemployment rate, total population, county and month by year fixed effects, and a county linear time trend | | | | | | | |
